# Structure of *Dictyostelium discoideum* telomeres. Analysis of possible replication mechanisms

**DOI:** 10.1371/journal.pone.0222909

**Published:** 2019-09-24

**Authors:** Javier Rodriguez-Centeno, Cristina Manguán-García, Rosario Perona, Leandro Sastre

**Affiliations:** 1 Instituto de Investigaciones Biomédicas CSIC/UAM, C/ Arturo Duperier, IdiPaz, C/Pedro Rico, Madrid, Spain; 2 CIBER de Enfermedades Raras (CIBERER), Madrid, Spain; Tulane University Health Sciences Center, UNITED STATES

## Abstract

Telomeres are nucleo-protein structures that protect the ends of eukaryotic chromosomes. They are not completely synthesized during DNA replication and are elongated by specific mechanisms. The structure of the telomeres and the elongation mechanism have not been determined in *Dictyostelium discoideum*. This organism presents extrachromosomal palindromic elements containing two copies of the rDNA, also present at the end of the chromosomes. In this article the structure of the terminal region of the rDNA is shown to consist of repetitions of the A(G)n sequence where the number of Gs is variable. These repeats extend as a 3’ single stranded region. The G-rich region is preceded by four tandem repetitions of two different DNA motifs. *D*. *discoideum* telomere reverse transcriptase homologous protein (TERTHP) presented RNase-sensitive enzymatic activity and was required to maintain telomere structure since *terthp*-mutant strains presented reorganizations of the DNA terminal regions. These modifications were different in several *terthp*-mutants and changed with their prolonged culture and subcloning. However, the *terthp* gene is not essential for *D*. *discoideum* proliferation. Telomeres could be maintained in *terthp*-mutant strains by homologous recombination mechanisms such as ALT (Alternative Lengthening of Telomeres) or HAATI (heterochromatin amplification-mediated and telomerase-independent). In agreement with this hypothesis, the expression of mRNAs coding for several proteins involved in homologous recombination was induced in *terthp*-mutant strains. Extrachromosomal rDNA could serve as substrate in these DNA homologous recombination reactions.

## Introduction

Telomeres are specialized protective nucleo-protein structures located at the terminal ends of the chromosomes [1]. In the absence of functional telomeres, the DNA at the edge of the chromosomes is recognized as damaged DNA and can be joined to other telomeres giving place to telomere-to-telomere fusions, resulting in genomic instability [2, 3]. In addition, unprotected telomeres initiate a cellular DNA damage response that can result in apoptosis or cellular senescence [4]. The importance of telomere homeostasis is shown by the existence of a number of human syndromes and diseases caused by excessive telomere shortening due to mutations in genes coding for proteins involved in telomere extension and protection [2, 3, 5].

The nucleotide sequence of the terminal telomeric DNA is related in most eukaryotic organisms. For example, telomeres in vertebrates consist of repetitions of the hexanucleotide sequence TTAGGG. In humans, these tandem repeats extend for 10–15 kb at the end of the chromosomes [6, 7]. In yeast, telomeres consist of imperfect repeats of the T-(G)_1-3_ (*S*. *cerevisiae*) [8] or TTAC(A)GG(G)_1-4_ (*S*. *pombe*) [9] motifs that extend for approximately 300 bp. Several species from the genera Allium present telomeres composed by repetitions of the CTCGGTTATGGG sequence [10].

The terminal DNA region is not blunt-ended and the 3’-end strand extends into a G-rich single-stranded region [11]. The G-rich strand folds back and invades the upstream DNA to form a tertiary structure, termed T-loop [12]. A protein complex interacts with the T-loop and telomeric repeats to form a heterochromatin structure required for telomere protection. In vertebrates, the protein complex is named shelterin complex [13] and mutations in the genes coding for some component are pathogenic, as mentioned above.

DNA replication at the telomeres cannot be completed by DNA polymerases and telomeres would be progressively shortened at each DNA replication during the cell cycle [14]. This process is reverted in most organisms by the telomerase complex that contains a reverse transcriptase enzyme and a RNA template that hybridizes with the telomeric DNA and is used for telomere elongation [15, 16].

However, some organisms present telomere structure and extension mechanisms very different from the ones described above [17]. One example is *Drosophila melanogaster* whose telomeres are formed by tandem repeats of Het-A, TAHRE and TART retrotransposons that extend for up to 12 kb in length [18]. Telomeres are extended by targeted transposition of these elements [19].

Alternative mechanisms of telomere extension have been observed in diverse organisms when they are devoid of telomerase activity [20]. Some cancer cells and yeasts activate a mechanism of alternative lengthening of the telomeres (ALT) based on DNA recombination [21, 22]. These cells are characterized by the presence of extrachromosomal telomeric DNA that can be used as substrate for homologous recombination. Given the repetitive sequence of the telomere, recombination can be unequal resulting in telomere elongation and also shortening [23]. As a consequence telomere size is very heterogeneous in these cells [24]. Another characteristic of these cells is the presence of nuclear aggregates known as ALT-associated Promyelocytic leukemia (PML) bodies or APBs [25]. These bodies contain telomeric DNA and telomere-binding proteins as well as proteins involved in DNA replication and recombination such as RAD50, RAD51, RAD52, RPA, MRE11, NBS1, BLM, WRN and SMC5/6 [21]. Additional mechanisms have been well studied in yeasts. *S*. *cerevisiae* cells lacking telomerase maintain linear chromosomes using the PAL(palindrome-dependent) mechanism [26]. In this case, telomeres are formed by large palindromes originated from smaller inverted repeats. *S*.*pombe*, in the absence of telomerase, can maintain linear chromosomes using a mechanism termed HAATI (heterochromatin amplification-mediated and telomerase-independent) [27]. Telomeres are replaced by repeated sequences, most frequently the rDNA, with a heterochromatic structure. The rDNA sequences are present at both sub-telomeric regions of chromosome III in wild type cells and spread to the telomere of all three HAATI chromosomes. Telomere structure and extension is maintained by continual homologous recombination among rDNA repeats present at each chromosomal end [20].

The amoeba *Dictyostelium discoideum* is a model organism characterized by its biological cycle with a phase of unicellular proliferation and another of development of a multicellular fruiting body initiated by cell aggregation. This organism has been the subject of extensive studies of biochemistry, cell migration, cell adhesion, morphogenesis and cell differentiation, among others [28–30]. The nucleotide sequence of *D*. *discoideum* genome was determined in 2005 and no canonical telomeric sequence was found at the ends of any of the six chromosomes [31]. Nuclei of *D*. *discoideum* contain about 100 copies of an 85 kb long palindromic extrachromosomal element that include two copies of the genes coding for the ribosomal RNAs [32, 33]. The terminal region of these elements was found at the end of several chromosomes, indicating that they provide the chromosome telomere regions [31]. However, the complete nucleotide sequence of the ends of the palindromic sequence and of the chromosomes could not be completely determined in the genome’s sequencing project due to the presence of multiple repeated sequences. Partial information on the sequence of the ends of the palindromes was reported by Emery and Weiner that showed the presence of irregular repeats of the (C)nT sequences preceded by four repetitions of a 29 bp long element [34]. In comparison, other *Dictyostelid* species subsequently sequenced such as *D*. *fasciculatum* of *P*. *pallidum* presented repetitions of the hexanucleotides TTAGGG and TAAGGG, respectively [35].

The analyses of the genome also showed that *D*. *discoideum* contained only one gene coding for a homologue to the telomere reverse transcriptase (*terthp*). However, this protein contained a long poly-Asparagine stretch inserted in the reverse transcriptase domain that could impair its enzymatic activity [31]. This insertion is not present in the other *Dictyostelid* species sequenced [35].

Because of these uncertainties, the structure of the end of the palindromic rDNA element, probable telomeric region of the *D*. *discoideum* chromosomes has been studied in this article. We report the presence of A(G)_n_ irregular repeats preceded by four repeats of two different sequence elements. Experimental evidence is provided for the presence of a 3’ single-stranded region at telomere ends similar to that of vertebrates. In addition, *D*. *discoideum* TERT-homologous protein (TERTHP) is shown to have putative telomerase activity and to be required to maintain telomere structure. However, the *terthp* gene is not essential for cell growth. *terthp*-mutant strains are viable but present altered telomere structure that we speculate could be maintained by DNA-recombination dependent mechanisms.

## Materials and methods

### Cell culture and transformation

*D*. *discoideum* cells were grown axenically in HL5 media under shaking (150 rpm) at 21°C. To assess growth rates, cells were suspended in HL5 media at a density of 3×10^5^ cells/mL and counted at different times of culture in triplicate using a hemocytometer. Cells were alternatively grown feeding on *Klebsiella aerogenes* over SM-agar plates. Cells were transformed by electroporation as previously described [36]. Transformed cells were selected by culture in HL5 media in the presence of 5 μg/ml blasticidin.

### Telomere amplification and nuclease digestions

The terminal sequence of the extrachromosomal rDNA element was amplified from genomic DNA (isolated according to [37]) either untreated or after treatment with nuclease S1 (Thermo Fisher Scientific. MA, USA) or exonuclease ExoI (Thermo Fisher Scientific) as recommended by the manufacturers. Primers were designed using the sequences published by Emery and Weiner [34]. The possible forward primers rDNA palindrome Forward-1 and -2 (Table 1) were designed according to the published proximal sequence and the possible reverse primers according to the distal sequence, rDNA palindrome Reverse-1 and -2 (Table 1). The primers *nola4* Forward and Reverse (Table 1) amplified a short region of the *nola4* gene and were used as control. PCR products were cloned into the p-GEMTeasy vector (Promega. WI, USA) using DH5α competent bacteria. The DNA isolated from several clones was sequenced. Later on, the telomere fragment was further extended in a second PCR reaction using a forward primer designed from the terminal sequences of the rDNA palindrome previously described (http://dictybase.org/) and a reverse primer designed from the sequence obtained for the products of the first PCR reaction. The primer sequences were: rDNA palindrome Forward-3 and Reverse-3, respectively (Table 1). PCR products were cloned and the DNA from several clones sequenced, as described above. The sequences obtained were aligned with MacVector software (MacVector. NC, USA)

**Table 1:** Oligonucleotides.

Gene	Orientation	Sequence (5’-3’)
*rDNA-palindrome*	Forward-1	*GGTTACGGTGGGAATCGAACC*
	Forward-2	*CCAATCGCCACCCTTAGCTTGG*
	Reverse-1	*GAGGGGGGAGGGGGGAGGG*
	Reverse-2	*CTCCCCCCTCCCCCCTCCC*
	Forward-3	*GGTATTGGATCGATAATATTGAGG*
	Reverse-3	*GGTTCGATTCCCACCGCTAACC*
*nola4*	Forward	*GGCTGCAGCTCCTAGTATTTCATCAAGAAATCC*
	Reverse	*GGAAGCTTTGTTGAAATTTCTTATTTTAAATAA*
*terthp*	Forward-arm-1	*GGGGTACCTAAAAGTCATCGATTCGTTGGAC*
	Reverse-arm-1	*GGAAGCTTGTTTCTATTGGAGGTTGATCTAG*
	Forward-arm-2	*GGCTGCAGAGGTGATGGTGATGATAATG*
	Reverse-arm-2	*GGGGATCCGGTAGTGGTAGTGGTAGTGG*
	Forward-test-1	*GTGGTGGTTATTTGAAGAGATC*
	Reverse-test-1	*CCAGCCAATTTGAACCAATCAAC*
*mre11*	Forward-1	*GGAAGCTTATGGAAGAAGAAGAAATAATTGAACC*
	Reverse-1	*CACCACCCAATAATACCATATC*
*rad51*	Forward-1	*GGAAGCTTATGGCATCAAGACAAAGACAAG*
	Reverse-1	*CAGAGATACCTTTGATACCTG*
*rad50*	Forward-1	*GAAACAAATTGAACTCGAATCATATG*
	Reverse-1	*CTTTATCTTGATTAGCTTTCTCTATATACTC*
*smc6*	Forward-1	*GGGGATCCATGTCCAAGAGGAAGTTAGGGC*
	Reverse-1	*CATCTTCACTTGAACCACTCTC*
*lmtrRNA*	Forward-1	*GGGTAGTTTGACTGGGGCGG*
	Reverse-1	*CACTTTAATGGGTGAACACC*
*26SrRNA*	Reverse-1	*GCAGTCACAACAGCGGGCTCC*

### Generation of *terthp—*Strains

Targeted disruption of the *terthp* gene in *D*. *discoideum* was accomplished using the pLPBLP vector, Cre-loxP system based, with resistance to blasticidin [38]. Targeting arms were amplified from *D*. *discoideum* (AX4) genomic DNA using the Herculase II fusion polymerase (Agilent technologies. CA, USA). The 5′ targeting arm was amplified using the primers: *terthp* Forward, arm-1 and Reverse, arm-1 (Table 1) which incorporated the *KpnI* and *HindIII* target sites to facilitate directional cloning into the vector. It covered the 313 bp upstream to the +1 and the first 736 bp of the gene. The 3′ targeting arm was amplified using the primers: *terthp* Forward, arm-2 and Reverse, arm-2 (Table 1) which incorporated *PstI* and *BamHI* sites. The 3´ arm covered the last 699 pb of the gene plus the next 303 pb. AX4 cells were electroporated with 10 μg of *KpnI/BamHI* (New England Biolabs. MA, USA) digested vector. Colonies resistant to blasticidin were collected. Genomic DNA was extracted using MasterAmp Buccal Swab DNA Extraction Solution (Epicentre. WI, USA). Gene disruption was validated by PCR reactions with two pair of primers shown in Table 1: 1) *terthp* Forward, test-1 and Reverse, test-1; 2) *nola4* Forward and Reverse (see results)

### Putative telomerase activity assay

Putative telomerase activity was measured using the TRAPeze RT Telomerase Detection Kit (Merck, Germany). Briefly, 1x10^7^ cells were collected by centrifugation, resuspended in 200 μL of 3-[(3-cholamidopropyl) dimethylammonio]-1-propanesulfonate (CHAPS) lysis buffer, incubated on ice for 30 min and centrifuged at 12000 g for 20 min at 4°C. 160 μL of supernatant was then transferred into a fresh tube and stored at -80°C until analysis. The TSR8 oligonucleotide, supplied with the kit, presents a sequence identical to the TS primer with an extension of 8 telomeric repeats AG (GGTTAG)_7_. This was used to generate an assay standard curve [39]. Serial dilutions were included in the analysis at levels of 0.4 amoles, 0.04 amoles, 0.004 amoles and 0.0004 amoles. A telomerase-positive cell extract provided with the kit was also included in the analysis, along with a ‘minus telomerase’ control (CHAPS buffer only), a no-template control (water only), and a heat-inactivated control for each sample (95°C for 20 min). RNase-treated samples were incubated 15 min at 37°C with 0.05 mg/mL of the enzyme. In addition, extracts from AX4 and *terthp* mutants were mixed and assayed for telomerase activity to determine if an inhibitor was present in the *terthp* mutant extract. Each experimental sample reaction contained the following: 5 μL of 5X TRAPEZE RT Reaction Mix, 0.4 μL of Titanium Taq DNA Polymerase (Takara, Japan), 12.1 μL of nuclease-free water and 7.5 μL of every sample (200 ng/ μL). PCR conditions for the assay were as follows: 1 cycle of 30°C for 30 min; one cycle of 95°C for 2 min; 45 cycles of 94°C for 15 s, 59°C for 60 s; and 45°C for 10 min with signal acquisition.

### Southern blot analyses

*D*. *discoideum* DNA (1 μg) was incubated with Bal31 exonuclease (NewEngland Biolabs, MA, USA) at 30°C for the times indicated in each experiment. Bal31 was inactivated by incubation at 65°C for 15 minutes and the DNA precipitated with ethanol. DNAs either treated with Bal31 or not, depending on the experiment, were digested with 10 unit of *NheI* (NewEngland Biolabs, MA, USA) for 2 hours at 37°C. Digested DNAs were electrophoresed in 1.2% agarose gels and transferred to Z-Probe membranes (BIO-RAD Laboratories, Inc. CA, USA) using 0.4 M NaOH. Oligonucleotides were labelled for 1 hour at 37°C using T4 polynucleotide kinase (Promega, Co. WI, USA) and ^**32**^P-γATP. Hybridization was made in 6xSSC, 1xDenhardt’s solution, 0.5% SDS and 100 μg/ml of calf thymus DNA at 37°C for 16 hours. Filters were washed in 6xSSC, 0.1% SDS at 37°C.

### Reverse transcription and quantitative PCR

Total RNA was obtained from 2x10^7^ *D*. *discoideum* cells using the TRI-reagent (Invitrogen, O, USA) according to the manufacturer’s instructions. cDNAs were generated from 2μg of RNA using random primers and M-MLV reverse transcriptase (Promega, Co. WI, USA) Quantitative PCR was made as previously described [40], using the Power SYBR PCR mix (Applied Biosystems. CA. USA) and the CFX96 Real-Time System (BioRad, CA, USA). The oligonucleotides used as primers for the amplification of each gene are described in Table1. A region of the large mitochondrial ribosomal RNA (*lmtrRNA*) was amplified as loading control. Relative gene expression quantification was calculated according to the comparative threshold cycle method (2^−ΔΔ*C*t^) and normalized to the amplification signal obtained for AX4 cells for each mRNA.

## Results

### 1. Structure of the terminal region of the *D*. *discoideum* rDNA palindromic element

**Fig 1:**
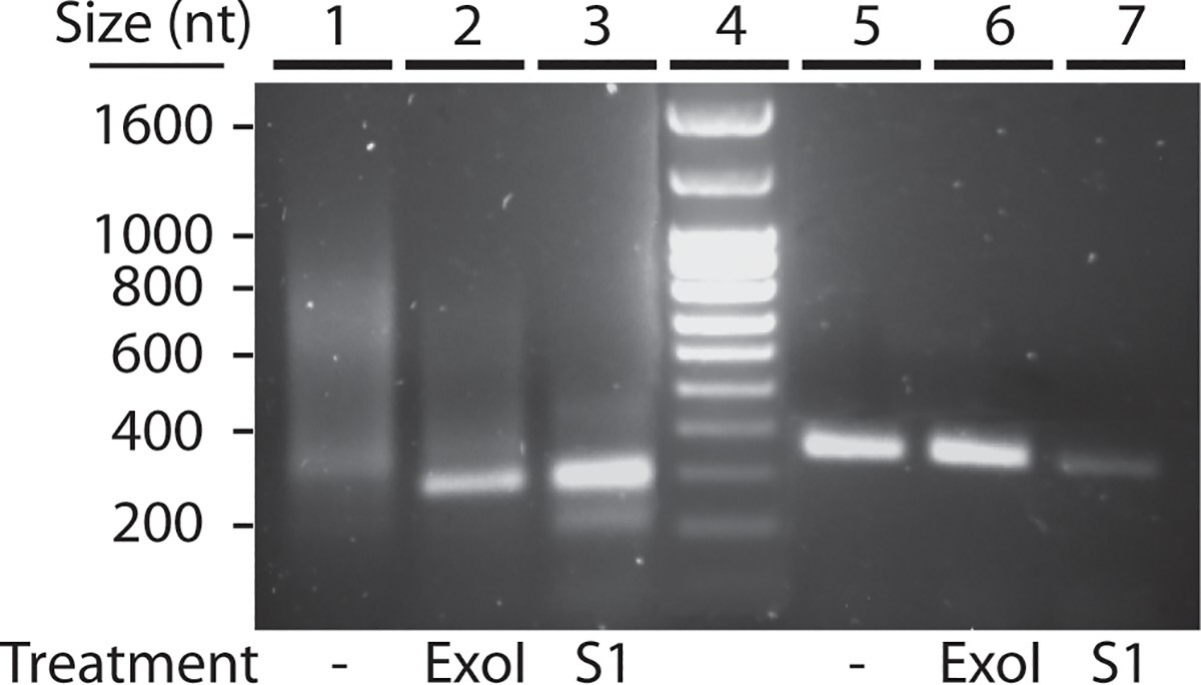
Analysis of the structure of the terminal region of the *D*. *discoideum* rDNA palindrome. Total DNA was extracted from *D*. *discoideum* AX4 cells and either non-treated (lanes 1, 5), digested with the 3’-5’ DNA single stranded exonuclease I (lanes 2, 6), or the DNA single-stranded specific nuclease S1 (lanes 3, 7). After treatment, the terminal region of the rDNA palindrome was amplified by PCR (lanes 1–3). A region of the *nola4* gene was also amplified as control (lanes 5–7). The migration of the Ladder VII molecular weight marker (Nzytech, Lisbon, Portugal) is shown in lane 4 and the size of the fragments indicated at the left of the picture.

Emery and Weiner [34] had described the sequence of two small DNA regions at the end of the rDNA palindrome, one composed by repetition of a T(C)_n_ motif where the number of cytidines was variable among repeats, and a fragment composed by 4 repeats of a 29 nucleotides long motif. However, the orientation of these sequences in relation to the palindrome was not determined in that article. Four oligonucleotides were designed to determine the relative orientation of these DNA fragments by PCR. Two primers were designed based on the T(C)_n_ sequence and two designed on the previous sequence of 4 repeats of a 29 nucleotides long motif (Table 1). The primer designed according to the published sequence of the long motif (Forward-1, Table 1) and the reverse primer designed in the T(C)_n_ sequence (Reverse-2) allowed the amplification of DNA fragments of variables sizes, from 300 to 800 bp (Fig 1, lane 1). Emery and Weiner had digested the DNA restriction fragment with S1 nuclease to blunt and clone it before sequencing which suggested that a single stranded overhanging region could exist at the end of the palindrome, as also happens in the telomeres of most organisms. To check for this possibility, DNA was digested with two single-strand specific nucleases, S1 nuclease and 3’-5’ exonuclease I, before the PCR reaction. The results showed that digestion with these nucleases resulted in the amplification of a single major band of 300 bp (Fig 1, lanes 2 and 3). In contrast, nuclease digestions did not alter the size of a control PCR product corresponding to the amplification of a single copy gene (*nola4*) (Fig 1, lanes 5–7). These results strongly support the presence of a single stranded overhang region at the end of the rDNA palindrome element.

**Fig 2:**
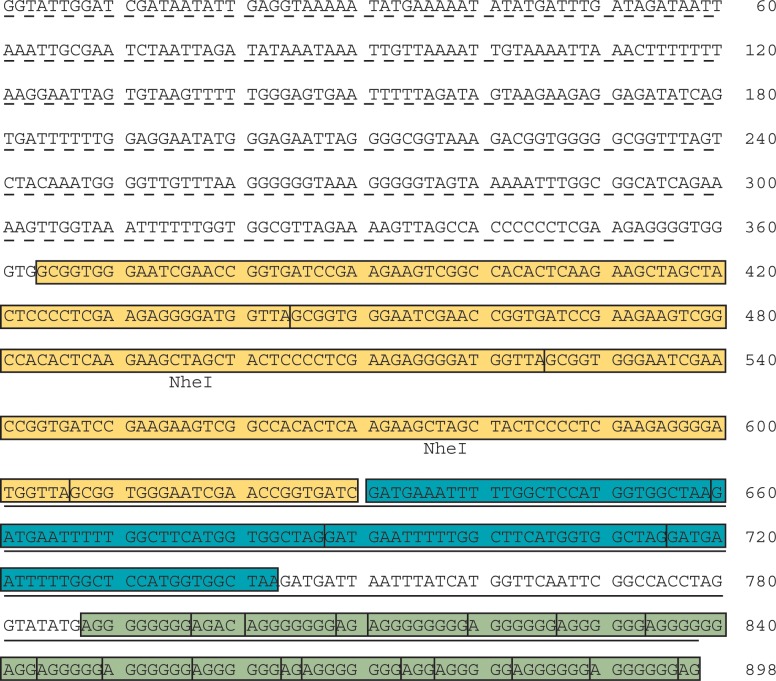
Nucleotide sequence of the terminal region of the *D*. *discoideum* rDNA palindrome. The nucleotide sequence of several PCR products corresponding to the terminal region of the rDNA palindrome was determined and the aligned sequence obtained is shown. The sequence underlined with a broken line (nucleotides 1 to 356) is coincident with the terminal fragment of the rDNA palindrome described in the Dictybase (http://dictybase.org/). The sequence underlined with a continuous line (nucleotides 602 to 898) corresponds to the sequences described by Emery *et al*, merged and reorganized. The nucleotide sequence corresponding to the more upstream repeats (repeats A in Fig 3) is shadowed in yellow, that of the middle repeats (repeats B in Fig 3) in blue, and the terminal repeats (C in Fig 3) in green. Individual repeats of each type are separated by vertical lines. The location of the *NheI* restriction sites used for Southern analyses is indicated underneath the respective sequences.

**Fig 3:**
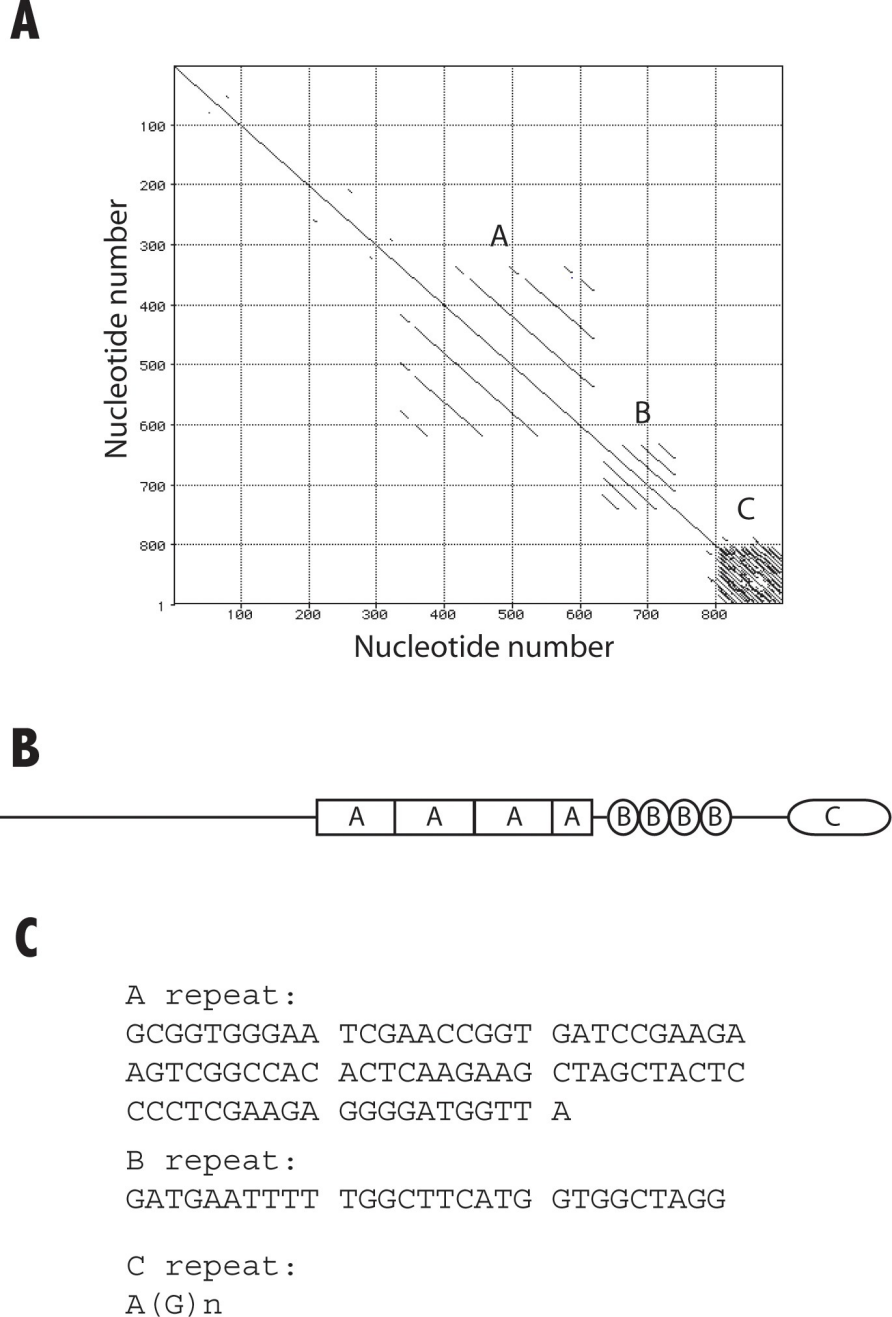
Structure of the terminal DNA sequence of the *D*. *discoideum* rDNA palindrome. The nucleotide sequence of the terminal region of the rDNA palindrome shown in Fig 2 was analyzed for the presence of repeated regions using a Matrix Plot. The results obtained are shown in panel A. The analysis showed the existence of three types of repeats, named A, B and C, as indicated in panel A and schematically represented in panel B. The sequence of a representative repetitive unit of each motif is shown in panel C. The terminal A(G)_n_ repeats correspond to motif C in this figure.

The PCR product amplified with the rDNA oligonucleotides was sequenced, as shown in Fig 2 (underlined sequence). The results showed the presence of 4 very similar repeats of 28–29 nucleotides in the same orientation described before [34] (Fig 2 and Fig 3, B repeats). However, the terminal region consisted of repetitions of a A(G)_n_ element, indicating the presence of a 3’ G-rich strand. This structure is similar to that present in the telomere region of most species, which contain a G-rich 3’ overhang [41].

Cloning of the PCR products obtained from untreated DNA allowed the identification of larger DNA fragments that were also sequenced. These fragments had up to four repetitions of a DNA element containing the 5’ oligonucleotide used for PCR amplification, as shown in the sequence of Fig 2, nucleotides 359 to 630. These four repeats are shown as A repeat in Fig 3. The DNA sequence has been submitted to the European Nucleotide Archive, accession ERZ502360.

Sucgang et al [33] had reported the sequence of the rDNA palindrome but the terminal region could not be completed. The more distal sequences determined corresponded to a fragment that could not be assembled to the rest of the palindrome, leaving a gap. To determine the relationship between the sequences determined by us and that described previously, as shown in the Dictybase (Dictybase.org), different oligonucleotides were designed from both regions and used for PCR amplification. The combination of two oligonucleotides (*rDNA* palindrome Forward-3 and Reverse-3, Table 1) allowed the amplification of a fragment of about 400 bp that overlapped both DNA sequences. The complete nucleotide sequence of the terminal region is shown in Fig 2. The region corresponding to the sequence previously published is underlined with a broken line. The orientation of this sequence is inverted with respect to that published at the Dictybase. Different oligonucleotides were also generated to try to fill the gap between this terminal fragment and the central palindrome region. However, these trials were unsuccessful, as also reported by Sucgang et al [33].

The structure of the DNA from the terminal region was analyzed for the presence of repetitive elements by doing a Matrix Plot that is presented in Fig 3, using MacVector software (MacVector, NC, USA). Three types of repetitive elements were found that have been named A to C from proximal to distal position. The A repeat is formed by three complete copies and a partial copy of a 81 bp long element shown in Fig 3. The B repeat is composed by 4 repetitions of the 28–29 bp long motif described by Emery and Weiner, limited by the conserved GATG sequence. The C repeat corresponds to the G-rich terminal region formed by repetition of the A(G)_n_ sequence where the number of Guanines is very variable, from one to eight (see Fig 2).

**Fig 4:**
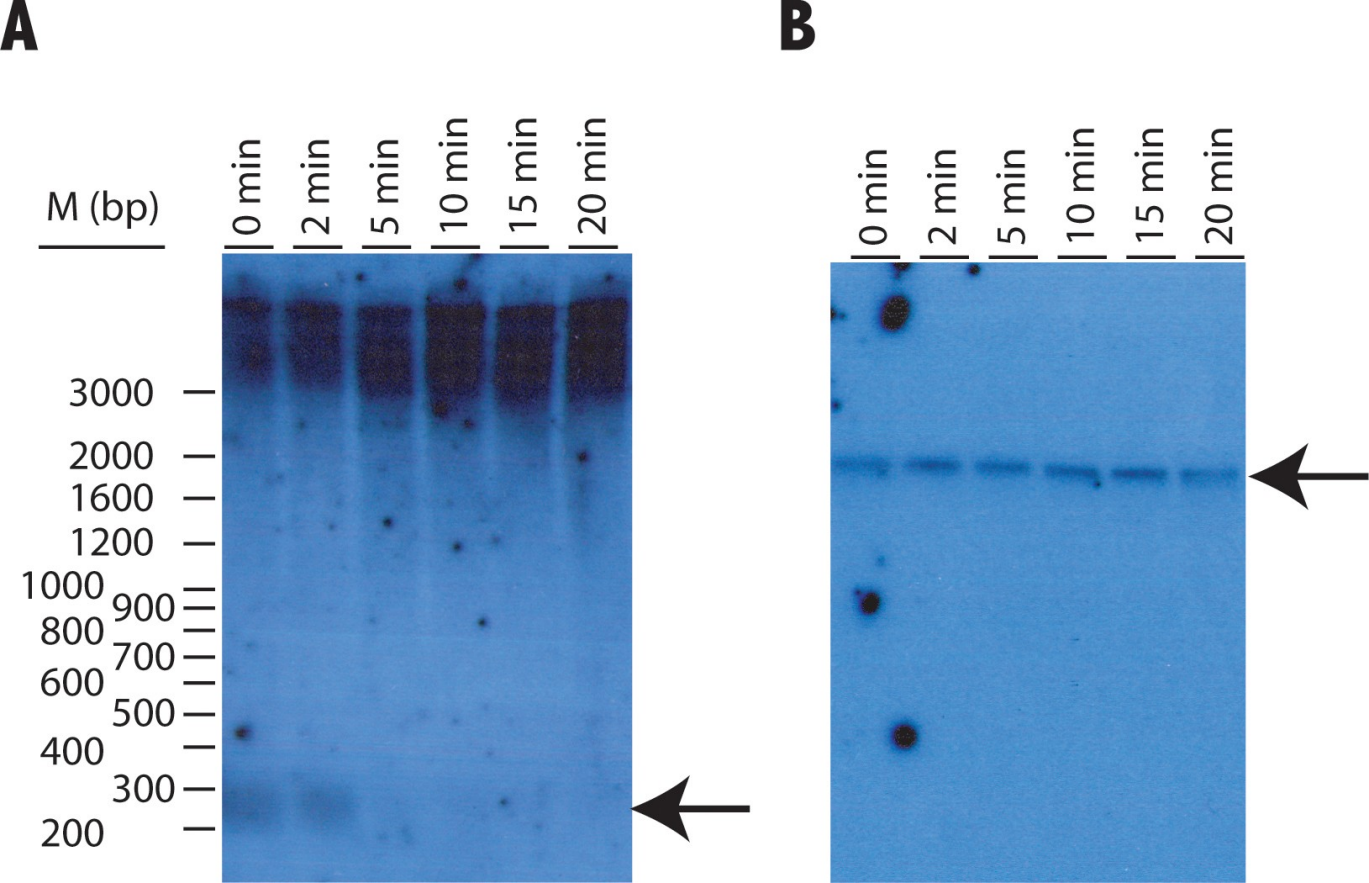
Southern blot analyses of Bal 31 sensitivity. Total DNA was isolated from AX4 cells and incubated with one unit of Bal31 exonuclease at 30°C for the periods of time indicated in the upper part of each lane. After this incubation, Bal31 exonuclease was inactivated and the DNA digested with the *NheI* restriction enzyme. Digested DNAs were blotted and hybridized to a oligonucleotide probe complementary to the A(G)n repeats (panel A) or a probe complementary to a region of the 26S rRNA coding gene (panel B). The migration of the Ladder VII molecular weight marker (Nzytech, Lisbon, Portugal) is shown to the left (M(bp) lane). Specific hybridizing bands are indicated by arrows in panels A and B.

The location of the characterized DNA at the terminal region was tested by digestion of *D*. *discoideum* DNA with the Bal31 exonuclease. Total DNA was digested with Bal31 for different periods of time. Treated DNA was subsequently incubated with the *NheI* restriction enzyme that generates a fragment of about 250 bp containing the T(G)n sequence (*NheI* sites are indicated in Fig 2). The digestion products were analyzed by Southern blot using the rDNA palindrome Reverse-2 primer (Table 1) as probe. Fig 4A shows that this probe hybridizes to a 250 bp-long *NheI* fragment and that this fragment is digested after more than 2 minutes of incubation with Bal31. In contrast, a probe complementary to the 26S rRNA gene, internal in the rDNA palindrome, is not digested after 20 minutes of Bal31 treatment (Fig 4B).

### 2. Studies on possible mechanisms of telomere replication in *D*. *discoideum*

**Fig 5:**
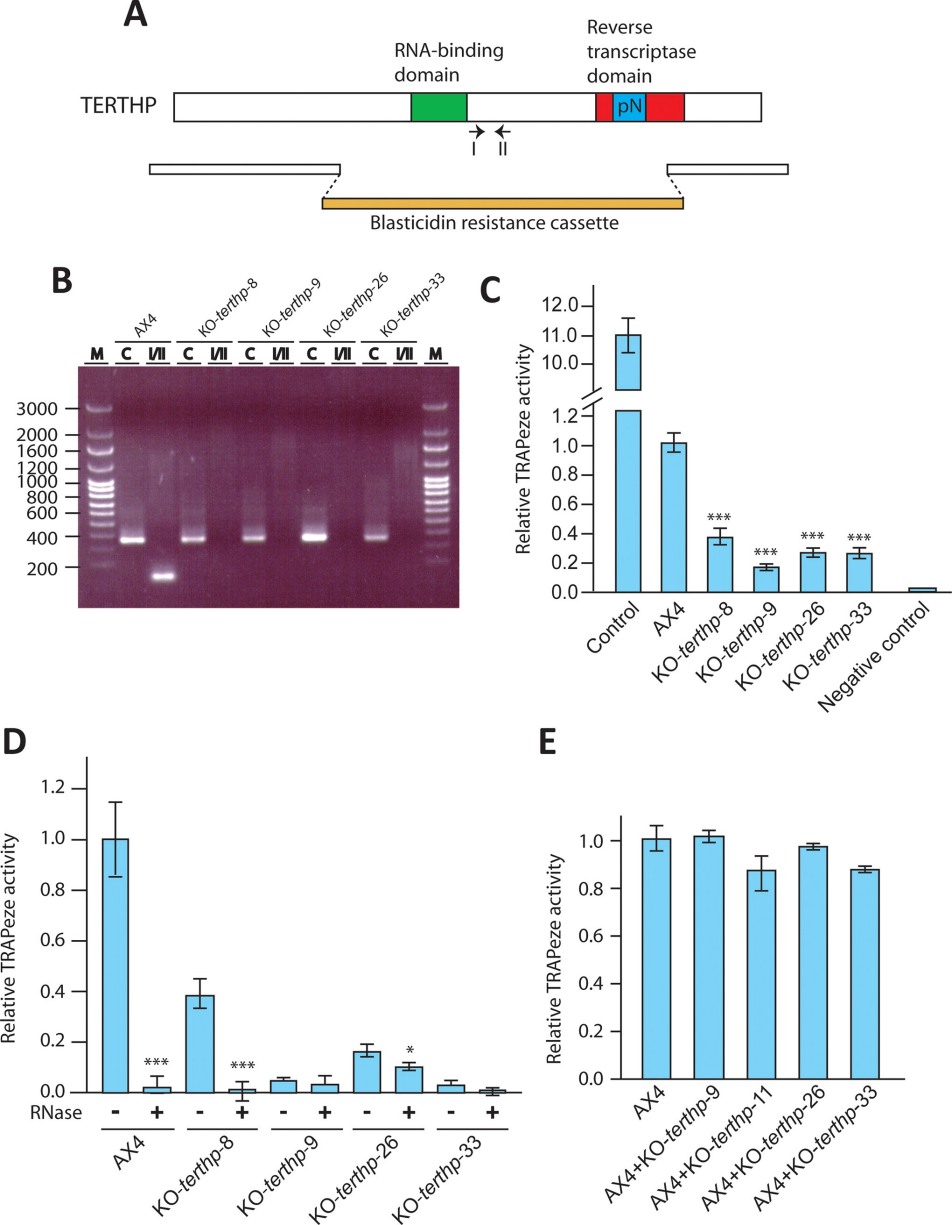
Generation of mutant strains where the *terthp* gene had been interrupted and determination of TRAPeze activity. Panel A shows a diagram of the *terthp* gene including the regions coding for the main functional domains of the protein and the poly-Asparagine stretch included in the reverse transcriptase domain. The gene organization resulting of the homologous recombination is shown underneath with the blasticidine-resistance cassette indicated in red colour. The insertion sites in the *terthp* gene, counted from the adenine of the initiation codon, correspond to nucleotides 736 and 3218. Panel B. Homologous recombination was tested by PCR using the pair of oligonucleotides I-II whose position on the gene is indicated under the diagram in panel A. The amplification products obtained for four mutant strains (KO-*terthp*-8, -9, -26 and -33) and AX4 wild type strain are shown (I/II lanes). Positive control amplifications were obtained using a pair of oligonucleotides specific for the *nola4* gene (C lanes). The migration of the Ladder VII molecular weight marker (Nzytech, Lisbon, Portugal) is shown in the M lanes and the size of some bands indicated to the left. Panel C. Determination of TRAPeze activity for a positive control extract, AX4, KO-*terthp*-8, KO-*terhpt*-9, KO-*terthp*-26, KO-*terthp*-33 strains and a negative control. The activity determined in the assay is shown in relation to that of the AX4 strain (value 1 in the Y axes). Standard deviations and statistical significance in comparison to AX4 activity are shown (*: p<0.05, **: p<0.01, ***: p< 0.001). Panel D. RNase sensitivity of TRAPeze activity. Extracts from the strains indicated in the lower part of the figure were treated with 0.05 mg/ml of RNaseA for 15 min at 37°C (+) or not (-). TRAPeze activity was determined as described in panel C. Panel E. Determination of TRAPeze activity in mixtures of different extracts. Extracts from AX4 and each of the mutant strains indicated in the lower part of the figure were mixed in equal amounts. The TRAPeze activity of the mixtures and of AX4 cells (AX4 column) was determined as described in panel C.

Telomeres are elongated by the telomerase complex at each round of DNA replication in most organisms, as mentioned in the introduction. *D*. *discoideum* contains a single gene coding for a protein highly homologous to the reverse transcriptase subunit of the telomerase complex, named TERT protein. The *D*. *discoideum* protein shares 23% of identical and 52% conserved residues with the human protein. The identity in conserved domains (schematically represented in Fig 5A) is of 22.7% in the RNA-binding and 31.8% in the reverse transcriptase domains. However, the protein presents an insertion of a long poly-Asparagine stretch in the catalytic domain that could inactivate the enzyme (Fig 5A) [31].

In order to test for the biological relevance of *D*. *discoideum* TERT-homologous protein (TERTHP), mutant strains with a large deletion of the gene (nucleotides 736 to 3218, Fig 5A) were generated by homologous recombination, as demonstrated by the absence of PCR amplification of an internal fragment of the *terthp* gene (Fig 5B). The size of plaques corresponding to mutant clones on the bacterial plates used for their isolation was smaller than that of wild-type clones, as shown in supplementary S1 Fig.

The possible telomerase activity of the wild-type (AX4) and *terthp*-mutant strains was determined using the TRAPeze RT Telomerase Detection Kit (Merck, Germany) that measures displacement of a fluorescent probe, and the results are shown in Fig 5C. One mammalian cells extract was used as positive control (provided by the kit) while the extraction buffer, CHAPS, and water were used as negative control. Extracts obtained from AX4 cells showed statistically significant higher activity than the extracts obtained from four different *terthp*-mutant strains even that lower than that of the mammalian cells control. Telomerase activity requires one RNA molecule as template and is, therefore, sensitive to RNase treatment of the extracts. This characteristic was tested by treatment of *D*. *discoideum* extracts with RNase A before the TRAPeze assay, as previously described [42]. This treatment drastically decreased the activity detected in AX4 extracts (Fig 5D) as also did with the control extract (data not shown). The activity detected in *terthp*-mutant’s extracts was also sensitive to RNase treatment although to different degrees depending on the strains (Fig 5D). The low activity of the mutants’ extracts could be due to the presence of some inhibitory molecule rather than to the inactivation of the *terthp* gene. To test for this possibility, equal amounts of AX4 and mutant extracts were mixed and analyzed for telomerase activity, as previously described [42]. The addition of mutant’s extracts did not affect AX4 activity, as shown in Fig 5E. Mutant extracts did not affect the activity of mammalian control extracts either (data not shown). These data are consistent with a putative telomerase activity of the TERTHP protein. However, *terthp* mutants should have no activity and they retained some. Therefore, the activity detected might not correspond to a telomerase reaction. Because of this reason, the protein studied will be referred to as TERT-homologous protein (TERTHP) and the mutant strains as *terthp*-mutants along the manuscript.

**Fig 6:**
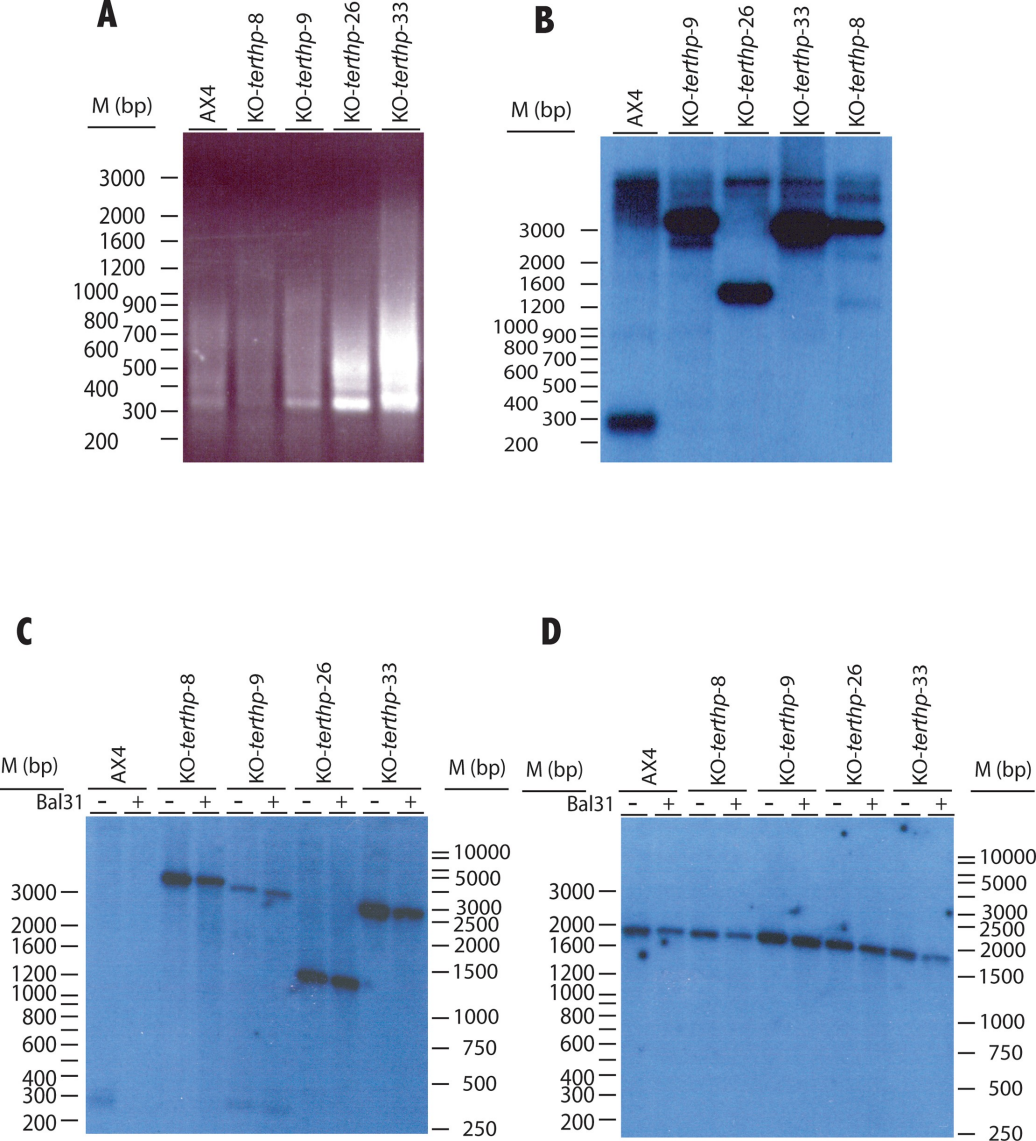
Determination of telomere structure in KO-*terthp* mutant strains. Panel A. Analyses of the size of the region containing A(G)_n_ repeats by PCR. Total DNA was isolated from the wild type (AX4) and *terthp*-mutant (KO-*terthp*-8, KO-*terthp*-9, KO-*terthp*-26 and KO-*terthp*-33) strains after 25 generations of growth and amplified by PCR using the same oligonucleotides described in Fig 1. Panel B. Southern blot analyses of total DNA digested with the *NheI* restriction enzyme and hybridized with a oligonucleotide probe complementary to the A(G)n repeats. Lanes are labelled as in panel A. Panels C and D. DNAs isolated from each strain were digested with 2 units of Bal 31 (+ Bal31 lanes) or not (- Bal31 lanes) for 60 minutes. Subsequently, DNAs were incubated with the *NheI* restriction enzyme and analyzed by Southern blot. The blot was hybridized with a oligonucleotide probe complementary to the A(G)_n_ repeats (panel C). Afterwards, blots were washed and hybridized to one oligonucleotide complementary to the 26S rRNA coding gene (panel D). Lanes are labelled as in panel A. In all panels, the migration of the Ladder VII molecular weight marker (Nzytech, Lisbon, Portugal) is shown to the left. The migration of the 1Kb ladder from Nippon Genetics Europe GmbH (Germany) is shown to the right of panels C and D.

The structure of the telomeric region of the mutant strains after 25 generations of growth was first analyzed by PCR amplification using the same pair of primers previously used in Fig 1. This assay indicated that similar fragments were amplified from AX4 wild type and *terthp* mutant strains (Fig 6A). The more significant difference was observed in the KO-*terthp*-33 mutant strain that showed larger and more heterogeneous amplification products than AX4 cells. Subsequent analyses were made by Southern blot after *NheI* digestion of the DNA (Fig 6B). This experiment showed a marked difference in the size of the digestion fragments that hybridized to the T(C)_n_ probe in AX4 WT and *terthp* mutant strains. Three of the mutant strains showed hybridization to a fragment of similar size (3 kb) while the other strain showed hybridization to a fragment of 1.3 kb. The change could correspond either to the variation of the nucleotide sequence driving to a change in the *NheI* restriction sites or to the addition of additional DNA to the end of the telomeric sequences. The first possibility was tested by molecular cloning and sequencing of the fragments previously obtained by PCR amplification from the KO-*terthp* strains shown in Fig 6A. The results obtained showed the conservation of the nucleotide sequence, including the *NheI* restriction sites (S1 Table). These data also showed the existence of small deletions and insertion (mostly Poly(A) stretches) in some of the amplified fragments.

The possible addition of additional DNA fragments at the DNA termini was tested by Southern blot analysis of total DNA incubated, or not, with Bal31 and digested with *NheI* (Fig 6C and 6D). The results obtained indicated that the fragment containing the A(G)n repeats is not sensitive to Bal31 digested for up to 60 minutes in the mutant strains, in contrast with the wild type AX4 strain, what would indicate that they are not located in DNA termini in these mutant strains. These results could be explained by the addition of DNA fragments to the telomere ends.

Mutant strains had been grown for 25 generations from their isolation to obtain the DNA and protein samples required for the experiments shown in Fig 5 (panels C, D, E) and Fig 6. The data obtained on telomere structure raised the question of their stability in the mutant strains. To study this problem, a subcloning strategy was approached [43]. Mutant strains were subcloned by plating them on bacteria and picking up individual colonies that were cultured for 25 generations (strains KO-*terthp*-8.1–9.1–11.1, -11.2, 26.1–26.2, -33.1 and wild-type strains AX4.1 and AX4.2). The subcloning process was repeated for four of the subclones by plating them and culturing individual clones for 25 generations (strains KO-*terthp*-8.1.A, -8.1.B, -9.1.A, -26.2.A and -33.1.A)

**Fig 7:**
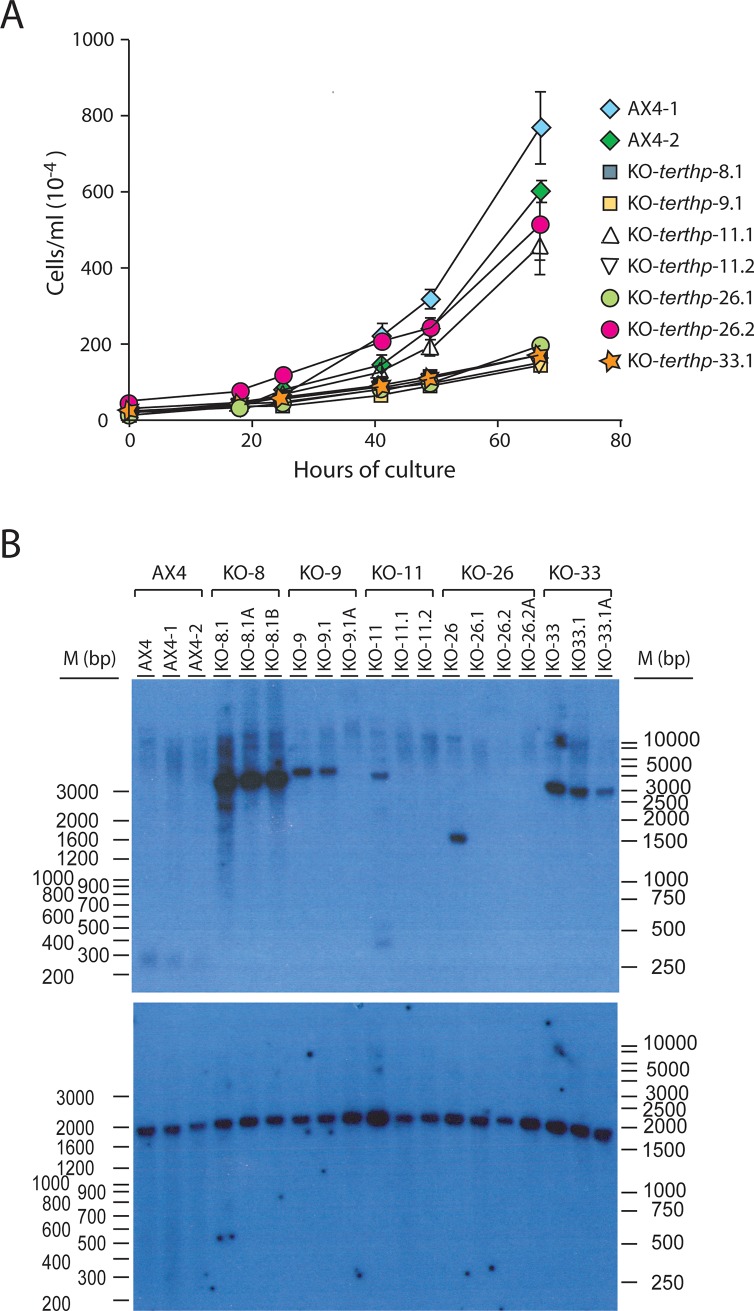
Characterization of the sublones generated from *D*. *discoideum terthp*-mutant strains. Panel A. Proliferation of *terthp*-mutant subclones. 3x10^5^ cells from wild-type AX4 and *terthp*-mutant subclones were cultured axenically. Cell concentration was determined at the times indicated in the X axes for each culture. Mean values and standard deviations are represented. Panel B. DNA was isolated from 3x10^7^ cells from the clones or subclones indicated in the upper part of the panel. Brackets indicated the original clones (shown above) and embrace the clones and subclones generated for each of them. DNAs were digested with the *NheI* restriction enzyme and analyzed by Southern blot. The upper part of the panel shows the hybridization obtained with the T(C)_n_ probe. The blot was washed and hybridized with a oligonucleotide complementary to the 26SrRNA coding gene (lower panel). The migration of the Ladder VII molecular weight marker (Nzytech, Lisbon, Portugal) is shown to the left of the panels and that of the 1 Kb ladder from Nippon Genetics Europe GmbH (Germany) to the right.

The proliferative capacity of the first generation of subclones was tested and compared to that of the AX4 subclones, as shown in Fig 7A. Subclones KO-*terthp*-11.1 and -26.2 proliferated to similar rates than AX4.1 and AX4.2 cells while the rest of mutant subclones presented reduced rates of proliferation, including subclones KO-*terthp*-11.2 and -26.1. These last subclones were isolated from the same strains as subclones KO-*terthp*-11.1 and -26.2 that showed higher proliferation what could indicate the divergent evolution of some subclones. The size of the plaques generated by the mutant strains on bacterial layers was also heterogeneous and generally smaller than that of the plaques formed by AX4 cells, as shown in supplementary S1 Fig.

Possible variations in telomere structure were tested by digestion of the isolated DNA with *NheI*, Southern blotting and hybridization to the previously described T(C)_n_ probe, as shown in the upper panel of Fig 7B. The results obtained showed that AX4 cells and some mutant strains, such as KO-*terthp*-8 and KO-*terthp*.33, presented a pattern of *NheI* digestion conserved during the subcloning process. Other mutant strains significantly changed their telomere structure and the main T(C)_n_ hybridizing fragment disappeared either in the first subcloning step (KO-*terthp*-11 and -26) or in the second step (KO-*terthp*-9). In contrast, the *NheI* digestion fragment that hybridizes to the 26SrRNA probe, also present in the rDNA palindromic repeat, was identical for AX4 and all the different subclones (Fig 7B, lower panel).

In the absence of telomerase activity, telomeres are elongated by an alternative mechanism such as ALT (alternative lengthening of telomeres) used in many biological systems including yeast devoid of telomerase activity [44] and human cancer cells [45] or the PAL (palindrome-dependent) mechanism used in yeast, as mentioned in the Introduction section. Both mechanisms are based on homologous recombination that is very efficient in *D*. *discoideum*. We, therefore, approached the study of the possible involvement of homologous recombination in telomere elongation in the mutant strains. There are some data that support this hypothesis such as the existence of a large number of rDNA palindromes in the nuclei that contain telomeric regions and could be the substrate for recombination. Their role would be equivalent to that of the extrachromosomal telomeric DNA found in cells that use the ALT mechanism [46].

**Fig 8:**
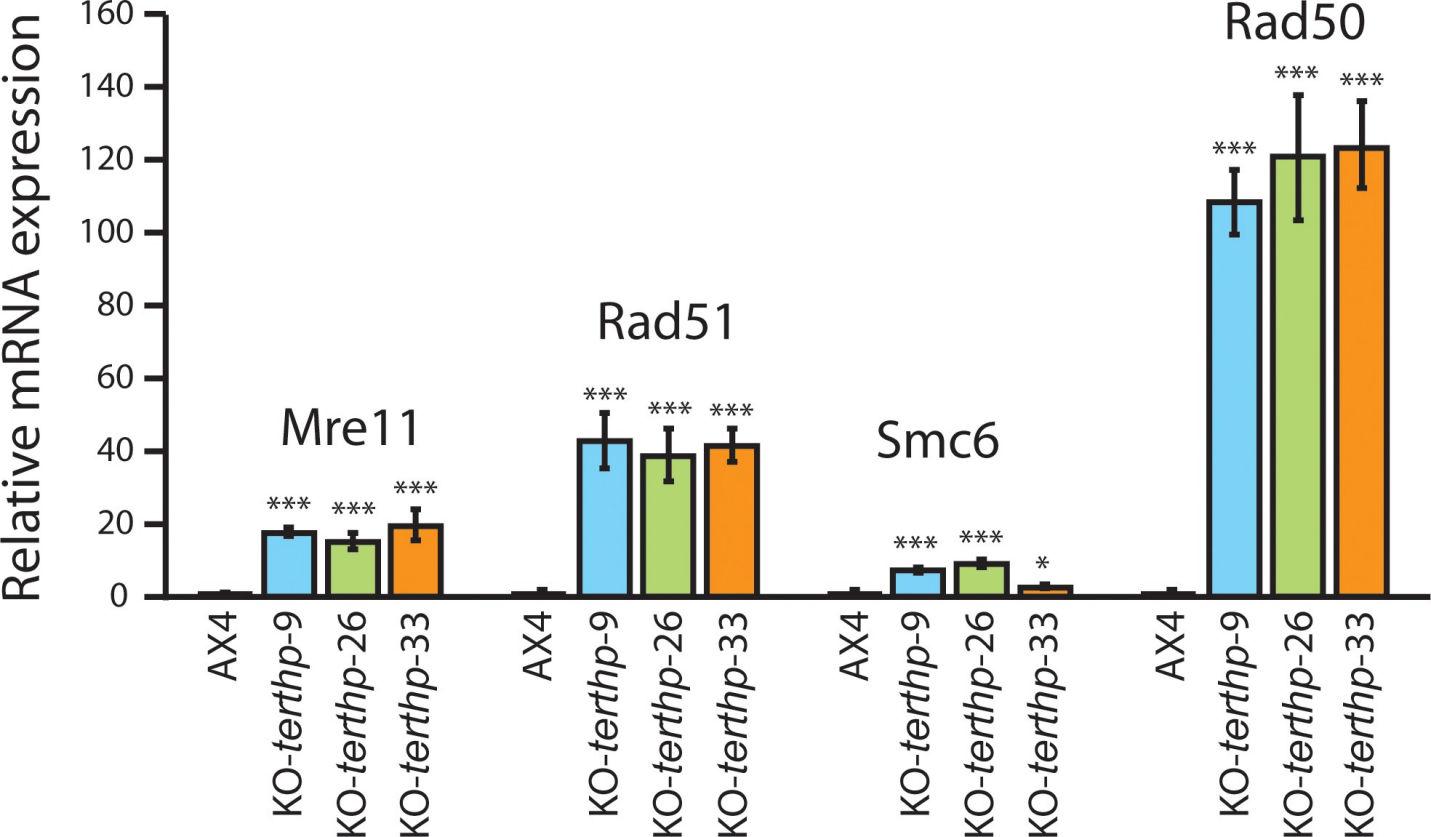
Expression of the homologous recombination-related genes *mre11*, *rad50*, *rad51* and *smc6* in *D*. *discoideum terthp*-mutant strains. Total RNA was isolated from the wild-type AX4 and the KO-*terthp*-9, KO-*terthp*-26 and KO-*terthp*-33 strains. RNA was reverse transcribed and the expression of the mRNAs coding for the proteins Mre11, Rad51, Smce6 and Rad50 analyzed by quantitative PCR. Relative expression compared to the expression of each mRNA in the AX4 strain is represented. The average relative expression, standard deviations and statistical significance are shown (*: p<0.05, **: p<0.01, ***: p< 0.001).

To study the possible induction of homologous recombination in *terthp* mutant strains, the expression of genes coding for several of the proteins involved in this pathway was determined. The relative level of expression of the genes *mre11*, *rad50*, *rad 51* and *smc6*, coding for proteins involved in DNA recombination, in three KO-*terthp* strains is shown in Fig 8 in relation to the expression in AX4 cells. These four genes showed a significant increase in expression in the mutant strains that is especially relevant for the *rad50* and *rad51* genes (100 and 40 times, respectively). These data support the hypothesis that homologous recombination could be induced in *D*. *discoideum terthp*-mutant strains.

## Discussion

The nucleotide sequence of the terminal region of the *D*. *discoideum* rDNA palindrome has been extended and organized, merging several partial sequences reported previously. We found that the terminal end of the rDNA element is formed by repeats of a consensus A(G)n sequence where the number of Guanines varies from one to eight. This sequence is preceded by four repetitions of a 28–29 bp long motif and 3 complete and a partial repetition of one 81 bp long motif. The terminal sequence contains a 3’ extension of G-rich single stranded DNA that can be digested by the single-strand S1 nuclease and single-strand 3’-exonuclease I. G-rich 3’ overhangs are common in most species. Similar to *D*. *discoideum*, telomere sequences of yeasts contain repetitions with different numbers of Guanines [8, 20]. This sequence was shown to present a terminal position on the DNA by Bal31-sensitivity studies. Southern blots also detected hybridization of the T(C)_n_ probe to higher molecular weight DNA that was not sensitive to Bal31 digestion and that could correspond to homologous sequences located in internal or subtelomeric regions.

Classical telomere sequences have not been found at the end of any of the 6 *D*. *discoideum* chromosomes, as mentioned in the introduction. Instead, genome-sequencing data indicate that terminal parts of the rDNA palindrome are present at the ends of all chromosomes [31]. Therefore, the terminal sequence of the rDNA palindrome here described is proposed to represent the telomeric region of all the chromosomes.

The presence of the G-rich 3’ overhang is important for the structure of the telomere since this region folds back to invade upstream DNA forming a loop (T-loop) required for telomere stability. In mammals, the telomere is the binding site of proteins of the shelterin complex, required for proper telomere structure. The G-rich overhang in *D*. *discoideum* could accomplish a similar protective role at the telomeres. The proteins of the mammalian shelterin complex are not conserved through evolution [47] and, accordingly, we have not been able to find *D*. *discoideum* homologous proteins. However, several telomere-binding proteins, such as mammalian TRF1 and TRF2 and proteins from other species have in common a myb DNA-binding domain [47, 48]. In *D*. *discoideum* there are several proteins that contain a myb domain that could play a role in telomere binding and protection. Among them MybAA and MybL present the highest similarity to human TRF1 and TRF2 proteins. The MybAA protein presents a 35% of identity to the Telobox DNA-binding domain of TRF1 and 44% to that of TRF2 (domains described by Poulet et al. [49]). MybL presents 39% of identity to TRF1 and 34% to TRF2 Telobox domains. Proteins binding to single-stranded telomere DNA are also found in many organisms and their main structural motif is the OB-fold (oligonucletotide/oligosaccharide binding fold), as is the case of the human POT1 protein [50]. [51]. There are many *D*. *discoideum* proteins with possible OB-folds but, among them, DDB_GO286375 presents a N-terminal region with 26% identity to human POT1 protein and also a second region with 24% identity to the C-terminal region of this human protein. Therefore, these proteins could be part of a *D*. *discoideum* telomere-binding complex similar to the shelterin complex.

The telomerase activity had not been studied in *D*. *discoideum* but the TERTHP protein was predicted to be inactive because of the presence of a large poly-Asparagine insertion in the reverse transcriptase domain. In the present article we have determined that *D*. *discoideum* TERTHP protein might be enzymatically active using the TRAPeze RT Telomerase Detection kit. The activity observed for AX4 extracts was significantly higher than that of *terthp*-mutant extracts, in support of the specificity of the activity detected. Telomerase use Telomere RNA (TR) as template but no homologous RNA molecule has been identified in *D*. *discoideum*. The possible dependence on a RNA template was tested by RNase digestion of the extracts before the TRAPeze reaction. This treatment completely abolished AX4 putative telomerase activity. *Terthp*-mutant extracts presented some activity that was also sensitive to RNase digestion. Since the only gene interrupted codes for a protein with homology to TERT in *D*. *discoideum*, this activity could correspond to a different polymerase enzyme that is also RNase-sensitive. The results of the TRAPeze assay could also be explained if mutant extracts would express telomerase inhibitors or some activity interfering with the TRAPeze assay. However, the addition of mutant extracts did not inhibit the putative telomerase activity of AX4 extracts or mammalian positive control extracts. Together, these data support the hypothesis that *D*. *discoideum terthp* gene codes for an active telomerase enzyme but there are some uncertainties due to the activity detected in the mutant strains that should completely lack it. One possible explanation is that the assay is detecting other enzymatic activities. By caution, the protein has been named TERT-homologous protein and the activity detected putative telomerase activity. In a recent study on *D*. *discoideum* TERTHP [52] the author did not detect telomerase activity in AX2 cells which could be due to different sensitivity of the assays used in both studies. In agreement with the data presented here, these authors also generated a *terthp*-mutant strain that was viable and only presented a developmental phenotype.

The role played by TERTHP in telomere maintenance is also supported in the present study by the observation that *terthp*-mutant strains have a modified telomere structure. The telomere DNA of the AX4 strain is present in the *terthp*-mutant strains but showed small deletions and insertion of poly(A) stretches and did not have a terminal position, as determined by the lack of sensitivity to Bal31 digestion. In contrast, the experimental results indicated that additional DNA was incorporated in the terminal region of the DNA.

Changes in telomere structure upon telomerase mutation have been observed in other organisms and are progressive [42, 53]. Telomere shortening is initially observed before the activation of alternative elongation mechanisms. In the present study this process of telomere attrition could not be observed but *terthp*-mutant colonies were initially very small, as compared to wild-type colonies, which might indicate an initial proliferation delay. Later on, mutant colonies proliferated in liquid culture and by the time the number of cells was sufficient for DNA analyses (25 generations) the telomere region had been restructured, as shown in Fig 5B. Subcloning and further growth of mutant strains by 25 or 50 additional generations has shown that telomere structure continued to evolve in three of the five mutant strains, as shown by Southern blot analyses in Fig 7B. Similar subculturing experiments did not detect changes in AX4 wild-type cells. The evolution and heterogeneity of mutant strains was also observed in their proliferation rate. While two of the subclones proliferate to similar rates as AX4 cells, others showed lower rates. There was not a complete correlation between growth rates and telomere structure. Subclones with higher proliferation capacity presented very altered telomere structure with no *NheI* fragment strongly hybridizing to the T(C)_n_ oligonucleotide probe. In contrast, other subclones with low hybridization signal were among those with lower proliferation rates. Actually, some subclones derived from the same original clone presented similar patterns of southern blot hybridization but different proliferation rates, as subclones 11.1/11.2 and 26.1/26.2. These data could indicate greater genome heterogeneity than the detected by the Southern blot analyses used in this study. Further genome-wide studies will be required to characterize the genetic evolution of *terthp*-mutant strains that could include DNA deletions, addition of DNA fragments and also telomere fusions. Additional studies would be also necessary to determine if TERTHP expression can restore telomere structure in the mutant strains.

*D*. *discoideum* chromosomes share some characteristics with HAATI yeasts chromosomes such as the presence of rDNA at telomeres [20]. In the case of *D*. *discoideum* the rDNA chromosomal copy is present in chromosome 4 and does not present a sub-telomeric location, as described in yeasts. Instead, rDNA copies could have been transferred to chromosomal ends from the extrachromosomal rDNA palindromic elements. These similarities allow us to consider the hypothesis that in *D*. *discoideum terthp*-mutant strains telomere length could be maintained by mechanisms similar to those used in HAATI yeasts.

In the absence of telomerase activity, telomeres are elongated by ALT (Alternative Lengthening of Telomeres) in many biological systems including yeast devoid of telomerase activity and 10–15% of human cancers. ALT is based on homologous recombination, a process that is very efficient in *D*. *discoideum* what could facilitate its implication in telomere elongation at *terthp*-mutant strains. Another data supporting this hypothesis is the existence of a large number of rDNA palindromes in the nuclei that contain telomeric regions and could be the substrate for recombination. Their role would be equivalent to that of the extrachromosomal telomeric DNA found in cells that use ALT. To further test this hypothesis we investigated the expression of the mRNAs coding for several proteins associated to the nuclear APB bodies, specific for ALT by RT-qPCR. Two of the proteins were Rad50 and Mre11, component of the mammalian MRN Complex (Mre11, Rad50, Nbs1) involved in homologous recombination and required for ALT [54, 55]. *D*. *discoideum* code for homologous proteins of Mre11 and Rad50 but not Nbs1. The expression of Rad 51, involved in homologous recombination [21], was also studied. The fourth protein studied was Smc6 that participates in DNA repair and in the DNA damage response as part of the Smc5/6 complex, is associated to APBs and required for ALT [56]. The expression of the mRNAs coding for these four proteins was significantly induced in the three *terthp*-mutant strains analyzed, as compared to wt AX4 cells. In the case of Rad50 and Rad51mRNAs, the induction was stronger (about 100 and 40 times, respectively). These results could indicate the induction of homologous recombination in these *terthp*-mutant strains. However, further experiments are required to determine the mechanism of telomere elongation in *D*. *discoideum terthp*-mutant strains,either ALT, HAATI or a different one. Previously mentioned studies on the genome-wide structure of *terthp*-mutant strains could also provide valuable information on the alternative elongation mechanisms activated in these strains.

In conclusion, the nucleotide sequence of the terminal region of the *D*. *discoideum* rDNA palindrome has been extended and organized, merging several partial sequences reported previously. The terminal end is formed by repeats of a consensus A(G)_n_ sequence where the number of Guanines varies from one to eight. This sequence is preceded by four repetitions of a 28–29 bp long motif and 3 complete and a partial repetition of a 81 bp long motif. The terminal sequence contains a 3’ extension of single stranded DNA. Telomere elongation in *D*. *discoideum* depends on the *terthp* gene, coding for the TERTHP protein with putative telomerase activity. However, *terthp*-mutant strains are viable and show modification in the structure of the telomeres that could be maintained by homologous recombination mechanisms such as ALT or HAATI.

## Supporting information

S1 Table Alignment of the nucleotide sequences of the products of PCR amplification of the terminal region of the KO-*terhpt* strains, shown in Fig 6A. PCR products were cloned in the pGEMT-Easy vector and the nucleotide sequence of the plasmid isolated from several colonies determined. Nucleotides sequences are aligned to that of the wild type AX4 strain shown in Fig 2 using the same colour code. Insertions and deletions with respect to AX4 are indicted in red. The *NheI* restriction site is underlined.
(DOC)

S1 Fig Culture of *terthp*-mutant strains on bacteria. Panel A. AX4 *D*. *discoideum* cells were transfected with the *terthp*-knockout vector and grown on *K*. *aerogenes* bacteria in SM-agar plates. The colonies formed after five days of culture are shown. Colonies indicated by arrows correspond to *terthp*-mutant strains as determined by PCR analyses. Panels B. Clones of *D*. *discoideum terthp*-mutant and AX4 wild type cells were picked up from bacterial plates (some of them shown in panel A) and cultured on liquid media for 25 generations. At this time, cells were plated in bacteria and individual clones picked up and cultured for 25 additional generations. Cells were plated in bacteria again and the clones formed are presented in this panel.
(DOC)
