# Supplementary material for: Structure of Dictyostelium discoideum telomeres. Analysis of possible replication mechanisms
Source: PLoS One. 2019 Sep 24;14(9):e0222909. doi: 10.1371/journal.pone.0222909 (PMC6759168; doi:10.1371/journal.pone.0222909)
Supplement: S1 Table — PCR products were cloned in the pGEMT-Easy vector and the nucleotide sequence of the plasmid isolated from several colonies determined. Nucleotides sequences are aligned to that of the wild type AX4 strain shown in Fig 2 using the same colour code. Insertions and deletions with respect to AX4 are indicted in red. The NheI restriction site is underlined. (DOC) [file pone.0222909.s001.doc]

**S1 Table. Alignment of the nucleotide sequences of the products of PCR amplification of the terminal region of the KO-*terhpt* strains, shown in Fig 6A.**

PCR products were cloned in the pGEMT-Easy vector and the nucleotide sequence of the plasmid isolated from several colonies determined. Nucleotides sequences are aligned to that of the wild type AX4 strain shown in Fig 2 using the same colour code. Insertions and deletions with respect to AX4 are indicted in red. The *NheI* restriction site is underlined.

Amplification products of the strains KO-*terthp*-8

AX4 GGTTAGCGGTGGGAATCGAACCGGTGATCCGAAGAAGTCGGCCACACTCAAGAAGCTAGC

KO8-5 GGTTAGCGGTGGGAATCGAACCGGTGATCCGAAGAAGTCGGCCACACTCAAGAAGCTAGC

KO8-3 GGTTAGCGGTGGGAATCGAACCGGTGATCCGAAGAAGTCGGCCACACTCAAGAAGCTAGC

KO8-1 ------------------------------------------------------------

AX4 TACTCCCCTCGAAGAGGGGATGGTTAGCGGTGGGAATCGAACCGGTGATCGATGAAATTT

KO8-5 TACTCCCCTCGAAGAGGGGATGGTTAGCGGTGGGAATCGAACCGGTGATCGATGAAATTT

KO8-3 TACTCCCCTCGAAGAGGGGATGGTTAGCGGTGGGAATCGAACCGGTGATCGATGAAATTT

KO8-1 ------------------------------------------------------------

AX4 TTGGCTCCATGGTGGCTAAGATGAATTTTTGGCTTCATGGTGGCTAGGATGAATTTTTGG

KO8-5 TTGGCTCCATGGTGGCTAAGATGAATTTTTGGCTTCATGGTGGCTAGGATGAATTTTTGG

KO8-3 TTGGCTCCATGGTGGCTAAGATGAATTTTTGGCTTCATGGTGGCTAGGATGAATTTTTGG

KO8-1 -------------------GATGAATTTTTGGCTTCATGGTGGCTAGGATGAATTTTTGG

AX4 CTTCATGGTGGCTAGGATGAATTTTTGGCTCCATGGTGGCTAAGATGATTAATTTATCAT

KO8-5 CTTCATGGTGGCTAGGATGAATTTTTGGCTCCATGGTGGCTAAGATGATTAATTTATCAT

KO8-3 CTTCATGGTGGCTAGGATGAATTTTTGGCTCCATGGTGGCTAAGATGATTAATTTATCAT

KO8-1 CTTCATGGTGGCTAGGATGAATTTTTGGCTCCATGGTGGCTAAGATGATTAATTTATCAT

AX4 GGTTCAATTCGGCCACCTAGGTATATGAGGGGGGGGAGACAGGGGGGGAGAGGGGGGGGA

KO8-5 GGTTCAATTCGGCCACCTAGGTATATGAGGGGGGG-AGACAGGGGGGGAGAGGGGGGAGG

KO8-3 GGTTCAATTCGGCCACCTAGGTATATGAGGGGGGG-AGACAGGGGGGGAGAGGGGGGAGG

KO8-1 GGTTCAATTCGGCCACCTAGGTATATGAGG------------------------------

AX4 GGGGGGAGGGGGGAGGGGGGAGGAGGGGGAGGGGGGAGGGGGGAGAGGGGGGGAGGAGGG

KO8-5 -------------AGGGGCACTAAAAA--AGGGGGGAGGGGGGAGGGGGGAG

KO8-3 GGGG—-AGGGGGGAGGGGTACTAAAAA--AGGGGG-AGGGGGGAGGGGGGAG

KO8-1 -----------------------------------------------

Amplification products of the strains KO-*terthp*-9

AX4 GGTTAGCGGTGGGAATCGAACCGGTGATCCGAAGAAGTCGGCCACACTCAAGAAGCTAGC

KO9-6 ------------------------------------------------------------

KO9-5 GGTTAGCGGTGGGAATCGAACCGGTGATCCGAAGAAGTCGGCCACACTCAAGAAGCTAGC

KO9-4 GGTTAGCGGTGGGAATCGAACCGGTGATCCGAAGAAGTCGGCCACACTCAAGAAGCTAGC

KO9-2 GGTTAGCGGTGGGAATCGAACCGGTGATCCGAAGAAGTCGGCCACACTCAAGAAGCTAGC

KO9-3 GGTTAGCGGTGGGAATCGAACCGGTGATCCGAAGAAGTCGGCCACACTCAAGAAGCTAGC

AX4 TACTCCCCTCGAAGAGGGGATGGTTAGCGGTGGGAATCGAACCGGTGATCGATGAAATTT

KO9-6 ---------------------GGTTAGCGGTGGGAATCGAACCGGTGATCGATGAAATTT

KO9-5 TACTCCCCTCGAAGAGGGGATGGTTAGCGGTGGGAATCGAACCGGTGATCGATGAAATTT

KO9-4 TACTCCCCTCGAAGAGGGGATGGTTAGCGGTGGGAATCGAACCGGTGATCGATGAAATTT

KO9-2 TACTCCCCTCGAAGAGGGGATGGTTAGCGGTGGGAATCGAACCGGTGATCGATGAAATTT

KO9-3 TACTCCCCTCGAAGAGGGGATGGTTAGCGGTGGGAATCGAACCGGTGATCGATGAAATTT

AX4 TTGGCTCCATGGTGGCTAAGATGAATTTTTGGCTTCATGGTGGCTAGGATGAATTTTTGG

KO9-6 TTGGCTCCATGGTGGCTAAGATGAATTTTTGGCTTCATGGTGGCTAGGATGAATTTTTGG

KO9-5 TTGGCTCCATGGTGGCTAAGATGAATTTTTGGCTTCATGGTGGCTAGGATGAATTTTTGG

KO9-4 TTGGCTTCATGGTGGCTAGGATGAATTTTTGGCTTCATGGTGGCTAGGATGAATTTTTGG

KO9-2 TTGGCTCCATGGTGGCTAAGATGAATTTTTGGCTTCATGGTGGCTAGGATGAATTTTTGG

KO9-3 TTGGCTCCATGGTGGCTAAGATGAATTTTTGGCTTCATGGTGGCTAGGATGAATTTTTGG

AX4 CTTCATGGTGGCTAGGATGAATTTTTGGCTCCATGGTGGCTAAGATGATTAATTTATCAT

KO9-6 CTTCATGGTGGCTAGGATGAATTTTTGGCTCCATGGTGGCTAAGATGATTAATTTATCAT

KO9-5 CTTCATGGTGGCTAGGATGAATTTTTGGCTCCATGGTGGCTAAGATGATTAATTTATCAT

KO9-4 CT----------------------------CCATGGTGGCTAAGATGATTAATTTATCAT

KO9-2 CT----------------------------CCATGGTGGCTAAGATGATTAATTTATCAT

KO9-3 CTTCATGGTGGCTAGGATGAATTTTTGGCTCCATGGTGGCTAAGATGATTAATTTATCAT

AX4 GGTTCAATTCGGCCACCTAGGTATATGAGGGGGGGGAGACAGGGGGGGAGAGGGGGGGGA

KO9-6 GGTTCAATTCGGCCACCTAGGTATATGAGGGGGGG-AGACAGGG-GGGGAGAGGGGGGAG

KO9-5 GGTTCAATTCGGCCACCTAGGTATATGAGGGGGGG-AGACAGG-----------------

KO9-4 GGTTCAATTCGGCCACCTAGGTATATGAGGGGGGG-AGACAGGG-GGGGAGAGGGGGGAG

KO9-2 GGTTCAATTCGGCCACCTAGGTATATGAGGGGGGG-AGACAGGGAGGGGGGAGGGGGGAG

KO9-3 GGTTCAATTCGGCCACCTAGGTATATGAGGGGGGG-AGACAGGGGGGGAGGGGGGAGGGG

AX4 GGGGGGAGGGGGGAGGGGGGAGGAGGGGGAGGGG

KO9-6 GAGGGGGGGAGAGGGGGGAGGGGGGAGGGGGGAG

KO9-5 ----------------------------------

KO9-4 GAGGGGAGGGGGGAGGGGGGAG------------

KO9-2 ----------------------------------

KO9-3 GGAG------------------------------

Amplification products of the strains KO-*terthp*-26

AX4 GGTTAGCGGTGGGAATCGAACCGGTGATCCGAAGAAGTCGGCCACACTCAAGAAGCTAGC

KO26-6 ------------------------------------------------------------

KO26-5 GGTTAGCGGTGGGAATCGAACCGGTGATCCGAAGAAGTCGGCCACACTCAAGAAGCTAGC

KO26-1 ------------------------------------------------------------

AX4 TACTCCCCTCGAAGAGGGGATGGTTAGCGGTGGGAATCGAACCGGTGATCCGAAGAAGTC

KO26-6 ---------------------GGTTAGCGGTGGGAATCGAACCGGTGATCCGAAGAAGTC

KO26-5 TACTCCCCTCGAAGAGGGGATGGTTAGCGGTGGGAATCGAACCGGTGATCCGAAGAAGTC

KO26-1 ---------------------GGTTAGCGGTGGGAATCGAACCGGTGATCCGAGGAAGTC

AX4 GGCCACACTCAAGAAGCTAGCTACTCCCCTCGAAGAGGGGATGGTTAGCGGTGGGAATCG

KO26-6 GGCCACACTCAAGAAGCTAGCTACTCCCCTCGAAGAGGGGATGGTTAGCGGTGGGAATCG

KO26-5 GGCCACACTCAAGAAGCTAGCTACTCCCCTCGAAGAGGGGATGGTTAGCGGTGGGAATCG

KO26-1 GGCCACACTCAAGAAGCTAGCTACTCCCCTCGAAGAGGGGATGGTTAGCGGTGGGAATCG

AX4 AACCGGTGATCGATGAAATTTTTGGCTCCATGGTGGCTAAGATGAATTTTTGGCTTCATG

KO26-6 AACCGGTGATCGATGAAATTTTTGGCTCCATGGTGGCTAAGATGAATTTTTGGCTTCATG

KO26-5 AACCGGTGATCGATGAAATTTTTGGCTCCATGGTGGCTAAGATGAATTTTTGGCTTCATG

KO26-1 AACCGGTGATCGATGAAATTTTTGGCTCCATGGCGGCTAAGATGAATTTTTGGCTTCATG

AX4 GTGGCTAGGATGAATTTTTGGCTTCATGGTGGCTAGGATGAATTTTTGGCTCCATGGTGG

KO26-6 GTGGCTAGGATGAATTTTTGGCTTCATGGTGGCTAGGATGAATTTTTGGCTCCATGGTGG

KO26-5 GTGGCTAGGATGAATTTTTGGCTTCATGGTGGCTAGGATGAATTTTTGGCTCCATGGTGG

KO26-1 GTGGCTAGGATGAATTTTTGGCTTCATGGTGGCTAGGATGAATTTTTGGCTCCATGGTGG

AX4 CTAAGATGATTAATTTATCATGGTTCAATTCGGCCACCTAGGTATATGAGGGGGGGGAGA

KO26-6 CTAAGATGATTAATTTATCATGGTTCAATTCGGCCACCTAGGTATATGAG----------

KO26-5 CTAAGATGATTAATTTATCATGGTTCAATTCGGCCACCTAGGTATATGAGGGGGGG-AGA

KO26-1 CTAAGATGATTAATTTATCATGG-------------------------------------

AX4 CAGGGGGGGAGAGGGGGGGGAGGGGGG

KO26-6 --------------------------

KO26-5 CAGGGGGGGGGAGGGGGGAGGGGGGAG

KO26-1 --------------------------

Amplification products of the strains KO-*terthp*-33

AX4 GGTTAGCGGTGGGAATCGAACCGGTGATCCGAAGAAGTCGGCCACACTCAAGAAGCTAGC

KO33-10 GGTTAGCGGTGGGAATCGAACCGGTGATCCGAAGAAGTCGGCCACACTCAAGAAGCTAGC

KO33-! GGTTAGCGGTGGGAATCGAACCGGTGATCCGAAGAAGTCGGCCACACTCAAGAAGCTAGC

KO33-13 GGTTAGCGGTGGGAATCGAACCGGTGATCCGAAGAAGTCGGCCACACTCAAGAAGCTAGC

KO33-18 GGTTAGCGGTGGGAATCGAACCGGTGATCCGAAGAAGTCGGCCACACTCAAGAAGCTAGC

KO33-16 GGTTAGCGGTGGGAATCGAACCGGTGATCCGAAGAAGTCGGCCACACTCAAGAAGCTAGC

KO33-7 GGTTAGCGGTGGGAATCGAACCGGTGATCCGAAGAAGTCGGCCACACTCAAGAAGCTAGC

KO33-3 GGTTAGCGGTGGGAATCGAACCGGTGATCCGAAGAAGTCGGCCACACTCAAGAAGCTAGC

KO33-2 GGTTAGCGGTGGGAATCGAACCGGTGATCCGAAGAAGTCGGCCACACTCAAGAAGCTAGC

AX4 TACTCCCCTCGAAGAGGGGATGGTTAGCGGTGGGAATCGAACCGGTGATCGATGAAATTT

KO33-10 TACTCCCCTCGAAGAGGGGATGGTTAGCGGTGGGAATCGAACCGGTGATCGATGAAATTT

KO33-! TACTCCCCTCGAAGAGGGGATGGTTAGCGGTGGGAATCGAACCGGTGATCGATGAAATTT

KO33-13 TACTCCCCTCGAAGAGGGGATGGTTAGCGGTGGGAATCGAACCGGTGATCGATGAAATTT

KO33-18 TACTCCCCTCGAAGAGGGGATGGTTAGCGGTGGGAATCGAACCGGTGATCGATGAAATTT

KO33-16 TACTCCCCTCGAAGAGGGGATGGTTAGCGGTGGGAATCGAACCGGTGATCGATGAAATTT

KO33-7 TACTCCCCTCGAAGAGGGGATGGTTAGCGGTGGGAATCGAACCGGTGATCGATGAAATTT

KO33-3 TACTCCCCTCAAAGAGGGGATGGTTAGCGGTGGGAATCGAACCGGTGATCGATGAAATTT

KO33-2 TACTCCCCTCGAAGAGGGGATGGTTAGCGGTGGGAATCGAACCGGTGATCGATGAAATTT

AX4 TTGGCTCCATGGTGGCTAAGATGAATTTTTGGCTTCATGGTGGCTAGGATGAATTTTTGG

KO33-10 TTGGCTCCATGGTGGCTAAGATGAATTTTTGGCTTCATGGTGGCTAGGATGAATTTTTGG

KO33-! TTGGCTCCATGGTGGCTAAGATGAATTTTTGGCTTCATGGTGGCTAGGATGAATTTTTGG

KO33-13 TTGGCTCCATGGTGGCTAAGATGAATTTTTGGCTTCATGGTGGCTAGGATGAATTTTTGG

KO33-18 TTGGCTCCATGGTGGCTAAGATGAATTTTTGGCTTCATGGTGGCTAGGATGAATTTTTGG

KO33-16 TTGGCTCCATGGTGGCTAAGATGAATTTTTGGCTTCATGGTGGCTAGGATGAATTTTTGG

KO33-7 TTGGCTCCATGGTGGCTAAGATGAATTTTTGGCTTCATGGTGGCTAGGATGAATTTTTGG

KO33-3 TTGGCTCCATGGTGGCTAAGATGAATTTTTGGCTTCATGGTGGCTAGGATGAATTTTTGG

KO33-2 TTGGCTCCATGGTGGCTAAGATGAATTTTTGGCTTCATGGTGGCTAGGATGAATTTTTGG

AX4 CTTCATGGTGGCTAGGATGAATTTTTGGCTCCATGGTGGCTAAGATGATTAATTTATCAT

KO33-10 CTTCATGGTGGCTAGGATGAATTTTTGGCTCCATGGTGGCTAAGATGATTAATTTATCAT

KO33-! CT----------------------------CCATGGTGGCTAAGATGATTAATTTATCAT

KO33-13 CTTCATGGTGGCTAGGATGAATTTTTGGCTCCATGGTGGCTAAGATGATTAATTTATCAT

KO33-18 CTTCATGGTGGCTAGGATGAATTTTTGGCTCCATGGTGGCTAAGATGATTAATTTATCAT

KO33-16 CTTCATGGTGGCTAGGATGAATTTTTGGCTCCATGGTGGCTAAGATGATTAATTTATCAT

KO33-7 CTT----------------------------CATGGTGGCTAAAAAAA--AAATT-----

KO33-3 CTTCATGGTGGCTAGGATGAATTTTTGGCTCCATGGTGGCTAAGATGATTAATTTATCAT

KO33-2 CTTCATGGTGGCTAGGATGAATTTTTGGCTCCATGGTGGCTAAGATGATTAATTTATCAT

AX4 GGTTCAATTCGGCCACCTAGGTATATGAGGGGGGGGAGACAGGGGGGGAGAGGGGGGGGA

KO33-10 GGTTCAATTCGGCCACCTAGGTATATGAGGGGGGG-AGACAGGGGGGGAGAGGGAGGGGG

KO33-! GGTTCAATTCGGCCACCTAGGTATATGAGGGGGGG-AGACAGGGGGGGAGAGGGGGGAGG

KO33-13 GGTTCAATTCGGCCACCTAGGTATATGAGGGGGGG-AGACAGGGGGGGAGAGGGGGGAGG

KO33-18 GGTTCAATTCGGCCACCTAGGTATATGAGGGGGGG-AGA---------------------

KO33-16 GGTTCAATTCGGCCACCTAGGTATATGAGGGGGGG-AGACAGGGGGGGAGAGGGGGGAGG

KO33-7 ---TCAAAAAAAAAAAAAAAAAAAAGGGGGGGGGGTACTAAAAAAAGGGGGGGAGGGGGG

KO33-3 GGTTCAATTCGGCCACCTAGGTATATGAGGGGGGG-AGACAGGGGGGGAGAGGGGGGAGG

KO33-2 GGTTCAATTCGGCCACCTAGGTATATGAGGGGGGG-AGACAGGGGGGGAGGGGGGAGGGG

AX4 GGGGGGAGGGGGGAGGGGGGAGGAGGGGGAGGGGGGAGGG

KO33-10 GAGGGGGGAG------------------------------

KO33-! AGGGGGGGCAGTAAAAAAGGGGGGAGGGGGGAGGGGGGAG

KO33-13 AGGGGGGGCAGTAAAAAAGGGGGGAGGGGGGAGGGGGGAG

KO33-18 ----------------------------------------

KO33-16 AGGGGGGGCAGTAAAAAAGGGGGGAGGGGGGAGGGGGGAG

KO33-7 AGGGGGGAG------------------------------

KO33-3 AGGGGGGGCAGTAAAAAAGGGGGGAGGGGGGAGGGGGGAG

KO33-2 GGAG------------------------------------
