# Supplementary material for: Structure of Dictyostelium discoideum telomeres. Analysis of possible replication mechanisms
Source: PLoS One. 2019 Sep 24;14(9):e0222909. doi: 10.1371/journal.pone.0222909 (PMC6759168; doi:10.1371/journal.pone.0222909)
Supplement: S1 Fig — Panel A. AX4 D. discoideum cells were transfected with the terthp-knockout vector and grown on K. aerogenes bacteria in SM-agar plates. The colonies formed after five days of culture are shown. Colonies indicated by arrows correspond to terthp-mutant strains as determined by PCR analyses. Panels B. Clones of D. discoideum terthp-mutant and AX4 wild type cells were picked up from bacterial plates (some of them shown in panel A) and cultured on liquid media for 25 generations. At this time, cells were plated in bacteria and individual clones picked up and cultured for 25 additional generations. Cells were plated in bacteria again and the clones formed are presented in this panel. (DOC) [file pone.0222909.s002.doc]

**S1 Fig . Culture of *terthp*-mutant strains on bacteria.** Panel A. AX4 *D. discoideum* cells were transfected with the *terthp*-knockout vector and grown on *K. aerogenes* bacteria in SM-agar plates. The colonies formed after five days of culture are shown. Colonies indicated by arrows correspond to *terthp*-mutant strains as determined by PCR analyses. Panels B. Clones of *D. discoideum* *terthp*-mutant and AX4 wild type cells were picked up from bacterial plates (some of them shown in panel A) and cultured on liquid media for 25 generations. At this time, cells were plated in bacteria and individual clones picked up and cultured for 25 additional generations. Cells were plated in bacteria again and the clones formed are presented in this panel.
